# Meditation treatment of Alzheimer disease and mild cognitive impairment: A protocol for systematic review

**DOI:** 10.1097/MD.0000000000019313

**Published:** 2020-03-06

**Authors:** Yunhui Chen, Jiayuan Zhang, Tiane Zhang, Liu Cao, Yanyan You, Chunjiang Zhang, Xinglong Liu, Qi Zhang

**Affiliations:** a Chengdu University of Traditional Chinese Medicine, Jin Niu District; b Sichuan Integrative Medicine Hospital, Wu Hou District, Chengdu, Sichuan, China.

**Keywords:** Alzheimer disease, meditation, mild cognitive impairment, systematic review

## Abstract

**Background:**

Growing body of scientific researches in recent years have suggested the promising effect of meditation on improving cognitive impairment of Alzheimer disease (AD) and mild cognitive impairment (MCI). This paper aims to provide a protocol for systematic review to evaluate the efficacy of meditation on cognition performance of patient with AD and MCI.

**Methods:**

The Cochrane Library, PubMed, EMBASE, Web of Science, the Chinese Biological Medicine Database, China National Knowledge Infrastructure, Wanfang database, and VIP information database will be searched systematically and electronically from establishment to March 2020. All published randomized controlled trials related will be included. Assessment of bias risk and data analyses will be implemented by Review Manager (V.5.3.5). The strength of the evidence will be assessed by the Grading of Recommendations Assessment, Development and Evaluation system.

**Results:**

A high-quality synthesis of current evidence of meditation for patient with AD and mild cognitive impairment will be provided in this study.

**Conclusion:**

This protocol of systematic review will be helpful for providing evidence of whether meditation is an effective and safe intervention for cognitive impairment of patient with AD and MCI.

**Ethics and dissemination:**

Ethical approval is unnecessary since this protocol is only for systematic review and does not involve privacy data or conduct an animal experiment. This protocol will be disseminated by a peer-review journal or conference presentation.

**Systematic review registration:**

PROSPERO CRD42019145932.

## 1 Introduction

Alzheimer disease (AD) is by far the most common form of dementia. In the US, it has become the sixth leading cause of death, an estimated 5.8 million people are living with AD and this figure is projected to increase to 14 million by 2050 as the population ages.^[1]^ Mild cognitive impairment (MCI) is considered a transitional stage between normal cognitive aging and dementia, an estimated 5 to 15% of individuals with MCI progressing to AD annually, 32% developed AD during 5 year's follow-up and up to 50% or more converted to AD eventually.^[2–5]^ Both AD and MCI are featured by progressive cognitive impairment, learning disability and memory loss and associated with significant public health concern and economic burden.^[6,7]^ However, by far effective therapies remain elusive and have become compelling challenges for the global societies to address.^[1]^ This highlights the urgent need for more preventive and therapeutic concepts and approaches.

In recent years, growing evidences have suggested that meditation, one of the non-pharmacological interventions, may offset or enhance cognitive function of patients with AD and MCI. Meditation, as an ancient mind-body practice, comprises a wide variety of meditation techniques, such as Metta, mantra, mindfulness, Zen, Kirtan Kirya, and Kundalini meditation practices. Although it is simple, economical, non-invasive and easy to learn and practice,^[8,9]^ mounting researches have demonstrated that meditation can improve multiple cognitive functions including attention, memory and executive ability;^[10–12]^ facilitate blood flow, oxygen delivery, and glucose utilization in the hippocampus, prefrontal cortex, and anterior cingulate gyrus;^[11,13–17]^ and positively affect brain structure relevant to cognition, including increasing cortical thickening and gray matter volume and offsetting age-related cortical thinning and gray matter loss.^[14,18,19]^

The effect of meditation on AD and MCI seems optimistic and promising, but a critical examination of the available evidence is still warranted. Systematic reviews of high quality randomized controlled trials (RCTs) offer the clearest evidence regarding the advantages of certain healthcare interventions. Herein, we would propose a protocol for a systematic review to evaluate the evidence of meditation's effects and safety on patients with AD and MCI.

## 2 Methods

### 2.1 Study registration

This systematic review protocol has been registered on PROSPTERO (www.crd.york.ac.uk/prospero/) with number CRD42019145932.

### 2.2 Selection criteria

#### 2.2.1 Types of studies

The studies included will be

1) RCTs that involved meditation or meditation combined with other therapy, while patients in control group were treated with medication alone or placebo, natural waiting-list;
2) the meditative intervention will include but not limit to Metta, mantra, mindfulness, Kundalini meditation, Zen, Zazen, transcendental meditation, and Kirtan Kirya practices;
3) minimum one cognitive outcome was reported with corresponding data.

Literature of non-randomized controlled trails, animal researches, case reports, reviews, conference proceedings and meta-analysis will be excluded.

#### 2.2.2 Types of patients

Patient with AD and MCI (as diagnosed using any recognized diagnostic criteria) will be included in the study. There will be no limitation on the gender, nation and duration of the disease.

#### 2.2.3 Types of interventions

RCTs involves meditation or meditation combined with other therapy or no treatment (waiting-listed) for comparison.

#### 2.2.4 Types of outcomes

The primary outcomes will include but not limit to the changes in the Mini-Mental State Examination, Alzheimer Disease Assessment Scale-cognitive subscale, Activities of Daily Living scale, Trial Making Test, Stroop Test, Digit Span, Hopkins Verbal Learning Test, Rey Auditory Verbal Learning Test, California Verbal Learning Test II, Rey Complex Figure Test, Clock-drawing Task, Lowenstein Occupational Therapy Cognitive Assessment and Boston Naming Test. Additional outcomes are improvement in biomarkers, effective rate and adverse event rate.

### 2.3 Search strategy

Studies published on the Cochrane Library, PubMed, EMBASE, Web of Science, the Chinese Biological Medicine Database, China National Knowledge Infrastructure, Wanfang database, and VIP information database will be searched from inception to March, 2020. The search keywords will be: AD, Alzheimer, Alzheimer disease, dementia mild cognitive impairment, senile dementia, meditation, Metta, mantra, mindfulness, Kundalini meditation, Zen, Zazen, transcendental meditation, and Kirtan Kirya. An equivalent translation of the search terms will be applied for Chinese and English database. Manual searches will also be conducted to identify the extra studies from the reference list. The search strategy for PubMed is presented in Table 1 and we will modify the strategy upon the requirement of other databases.

**Table 1:**
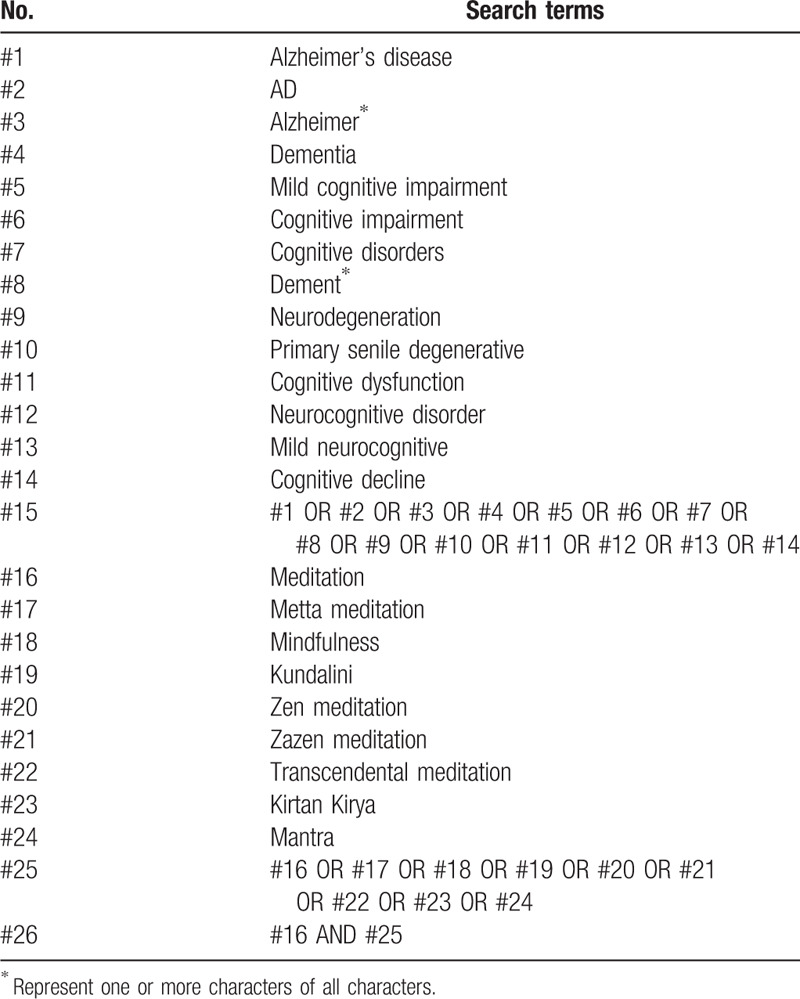
Search strategy for the PubMed.

### 2.4 Data collection and analysis

#### 2.4.1 Study selection and data extraction

**Figure 1:**
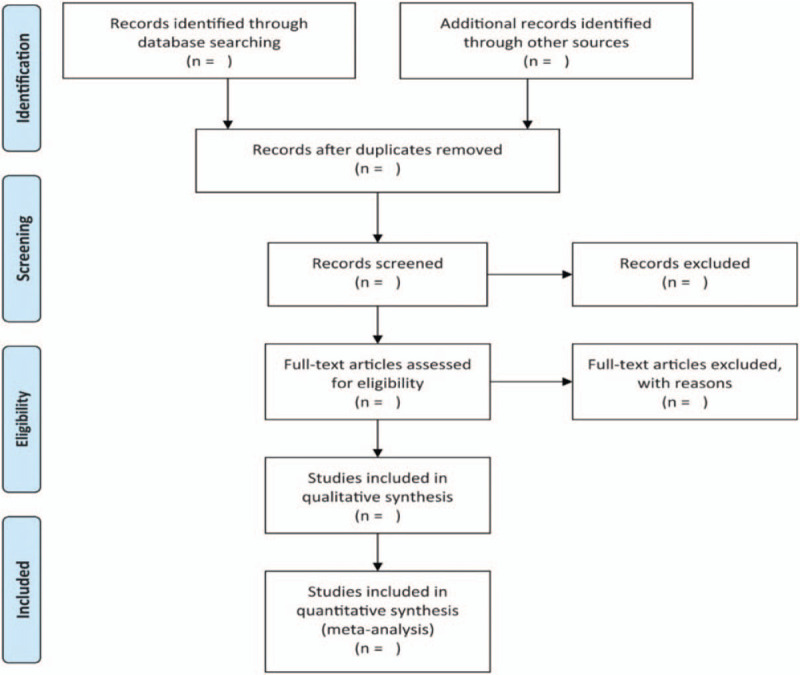
Flowchart of study selection.

Literature screening, study selection, and data extraction will be performed by 2 reviewers. The data extraction will be conducted in a standard form, which included characteristics of the study type, patient, meditative intervention details, outcomes, and adverse events. Disagreements will be solved by discussion until a consensus is reached. The data will be recorded in an excel spreadsheet. If the data provided in the research is unclear, missing or difficult to extract reliably, the corresponding author will be contacted for clarification. The flow diagram of study selection is shown in Figure 1.

#### 2.4.2 Assessment of risk of bias in included studies

The risks of bias will be assessed according to the Cochrane Handbook Version 5.1.0. by 2 reviewers. The following factors will be assessed: random sequence generation, allocation concealment, blinding of participants and personnel, blinding of outcome assessment, incomplete outcome data, selective reporting, and other bias. Third-party experts will be consulted for disagreement. A funnel plot will be conducted to assess publication bias when the included studies are more than 10.

#### 2.4.3 Data analysis

The systematic review of the literature will be performed based on meditative intervention approaches, measurement outcomes, the length of intervention and heterogeneity levels, and so on. Effective rate will be calculated by odds risk. Heterogeneity of the data will be assessed by Q-test and *I*^2^ statistic. Fixed-effect model will be applied when heterogeneity is low (*I*^2^ < 50%), random-effects model for moderate heterogeneity (50%–75%). While the heterogeneity is significantly high (*I*^2^ > 50%), subgroup analysis or descriptive analysis will be performed.

#### 2.4.4 Subgroup analysis and sensitivity analysis

If the necessary data are available, subgroup analyses will be conducted for different meditative approaches, duration, patient gender (Male/Female), primary outcomes and so on. After the quality assessment of the included literature, if there are possible low-quality studies, sensitivity analysis will be required. We will observe fluctuation of termination by changing the genre of research (incorporating or excluding a study) and reanalyze the simulated missing data.

#### 2.4.5 Grading the quality of evidence

The strength of the evidence for primary outcomes will be assessed by the Grading of Recommendations Assessment, Development and Evaluation (GRADE) into very low, low, moderate or high level.

## 3 Discussion

AD and MCI are both age-related cognitive disorders and clinically featured by cognitive impairment, learning disability and memory dysfunction. Currently, pharmacologic treatments can only temporarily improve symptoms and fail to restore the damage of the neurons. While non-pharmacologic therapies are frequently used to maintain or enhance the cognitive function, activities of daily life and overall quality of life.^[1]^ Among them, meditation is defined as “an intentional and self-regulated focusing of attention that aims to relax and calm the mind and body”^[20]^ and its impact on AD and MCI has been recently a topic of scientific interest. It is considered a cognition-stimulating activity, requires no specialized equipment, and is easy to practice with favorable long-term adherence.^[21]^ Meditation may hold considerable promise for improving cognition and related outcomes in patients with AD and MCI. Herein, this systematic review aims to assess the general effectiveness and safety of meditation in improving cognitive performance of AD and MCI, high-quality RCTs with no limits on language will be extracted and synthesized to establish a more convincing evidence for the clinicians and investigators in the field of geriatric sciences and contemplative sciences.

However, despite all the efforts made, there are some limitations. For instance, factors such as various types, frequency and duration of meditation intervention, gender and age of patients, as well as duration of AD and MCI disease may cause moderate to high heterogeneity. We should be aware of these potential factors so as to plan and power our study appropriately.

## Author contributions

**Conceptualization:** Yunhui Chen, Jiayuan Zhang.

**Data curation:** Jiayuan Zhang, Xinglong Liu, Yunhui Chen, Tiane Zhang.

**Formal analysis:** Jiayuan Zhang, Yunhui Chen, Yanyan You, Chunjiang Zhang.

**Funding acquisition:** Yunhui Chen, Xinglong Liu.

**Investigation:** Jiayuan Zhang, Xinglong Liu, Liu Cao, Yunhui Chen.

**Methodology:** Yunhui Chen, Jiayuan Zhang, Xinglong Liu.

**Project administration:** Yunhui Chen, Jiayuan Zhang, Tiane Zhang.

**Supervision:** Tiane Zhang, Qi Zhang.

**Validation:** Liu Cao, Chunjiang Zhang, Yunhui Chen, Xinglong Liu.

**Writing – original draft:** Yunhui Chen, Jiayuan Zhang.

**Writing – review & editing:** Yunhui Chen, Jiayuan Zhang, Xinglong Liu.
